# Gamification of the National Institutes of Health Stroke Scale (NIHSS) for simulation training—a feasibility study

**DOI:** 10.1186/s41077-023-00245-4

**Published:** 2023-02-22

**Authors:** Astrid Karina V. Harring, Jo Røislien, Karianne Larsen, Mona Guterud, Helge Fagerheim Bugge, Else Charlotte Sandset, Dorte V. Kristensen, Maren Ranhoff Hov

**Affiliations:** 1 Department for Prehospital Education and Research, OsloMet – Oslo Metropolitan University, P.O. Box 4, St. Olavs plass, Oslo, NO-0130 Norway; 2 Department of Research and Development, the Norwegian Air Ambulance Foundation, Oslo, Norway; 3 Department of Quality and Health Technology, University of Stavanger, Stavanger, Norway; 4 Division of Prehospital Services, Oslo University Hospital and University of Oslo, Oslo, Norway; 5 Faculty of Medicine, Institute of Clinical Medicine, University of Oslo, Oslo, Norway; 6 Department of Neurology, Oslo University Hospital, Oslo, Norway; 7 Department of Nursing and Health Sciences, University of South-Eastern Norway, Drammen, Norway

**Keywords:** Education, EMS, Gamification, Mobile Application, Neurology, Prehospital, Simulation

## Abstract

**Background:**

Training prehospital personnel in identifying patients with acute stroke is key to providing rapid treatment. This study aimed to investigate whether game-based digital simulation training is a feasible alternative to standard in-person simulation training.

**Methods:**

Second-year paramedic bachelor students at Oslo Metropolitan University in Norway were invited to participate in a study to compare game-based digital simulation (intervention) to standard in-person training (control). For 2 months, students were encouraged to practice the NIHSS, and both groups logged their simulations. Then, they performed a clinical proficiency test, and their results were assessed using a Bland-Altman plot with corresponding 95% limits of agreement (LoA).

**Results:**

Fifty students participated in the study. Individuals in the game group (*n* = 23) spent an average (SD) of 42:36 min (36) on gaming and performed 14.4 (13) simulations on average, whereas the control group (*n* = 27) spent 9:28 min (8) simulating and performed 2.5 (1) simulations. Comparing time variables collected during the intervention period, the mean time for each simulated assessment was significantly shorter in the game group (2:57 min vs. 3:50 min, *p* = 0.004). In the final clinical proficiency test, the mean difference from the true NIHSS score was 0.64 (LoA: − 1.38 to 2.67) in the game group and 0.69 (LoA: − 1.65 to 3.02) in the control group.

**Conclusion:**

Game-based digital simulation training is a feasible alternative to standard in-person simulation training to acquire competence in NIHSS assessment. Gamification seemed to give an incentive to simulate considerably more and to perform the assessment faster, with equal accuracy.

**Trial registration:**

The study was approved by the Norwegian Centre for Research Data (reference no. 543238).

## Introduction

Timely prehospital identification of patients with suspected acute stroke is essential to ensure rapid treatment and improve outcomes [1, 2]. Reducing misdiagnosis and prehospital delay by training prehospital personnel and utilising emerging technologies is recommended by the European Academy of Neurology and the European Stroke Organisation to increase the number of patients receiving acute treatment [3]. In the emergency medical services (EMS) in Norway, as in many other countries worldwide, the crude Face Arm Speech Time (FAST) test is the recommended tool to detect stroke symptoms [2, 4]. The National Institutes of Health Stroke Scale (NIHSS) is the most common stroke scale used in-hospital and recent studies have shown that paramedics can be trained in the full NIHSS, to detect and recognise symptoms of any type of stroke [2, 5, 6]. It also provide paramedics and stroke physicians with a common language along the stroke care chain [2, 7].

As part of the Paramedic Norwegian Acute Stroke Prehospital Project (ParaNASPP), an e-learning program with digital classroom training [7] and a mobile application that guides the paramedic through the NIHSS assessment have been created [8, 9]. However, frequent training is necessary to maintain clinical skills and this is often difficult due to infrequent exposure to stroke patients.

Applying principles from game theory like rapid decision-making based on limited information has been proposed for acute stroke care [10]. Gamification in simulation training is applying game mechanisms into non-game contexts [11] and has become popular in educational settings due to its engaging nature [12–15]. NIHSS has the potential for gamification due to the quantifiable and objective measures [16, 17], enabling standardised simulation training for paramedics without the risk of mistreatment of patients. In this study, we have developed a digital simulation game based on the NIHSS assessment: a smartphone application called GameStroke. The objective of this study was to assess the feasibility and agreement between game-based simulation training compared to traditional in-person simulation.

## Method

### GameStroke

**Fig. 1:**
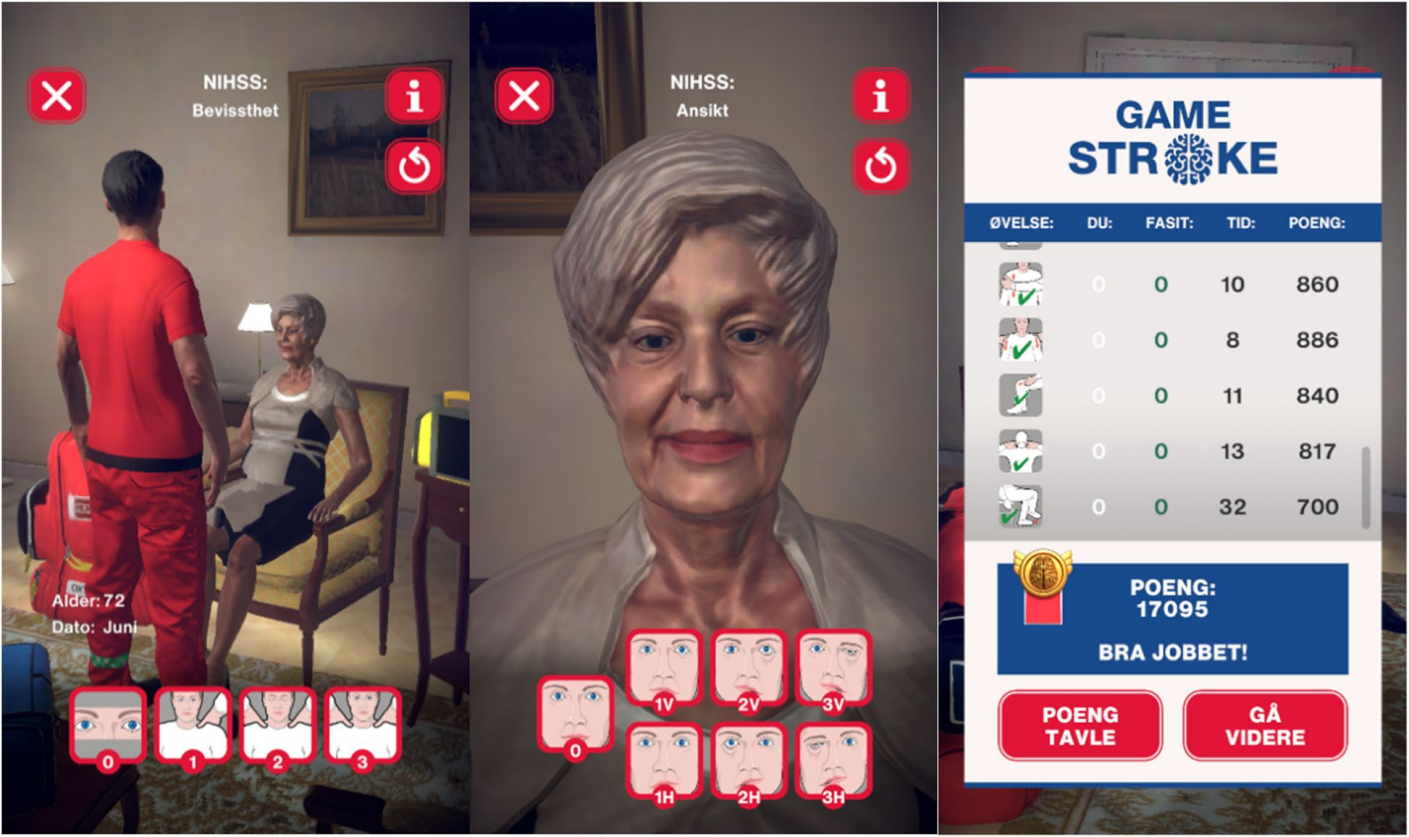
Examples of pictograms of the NIHSS assessment used in GameStroke

GameStroke was developed by the Norwegian Air Ambulance Foundation in collaboration with stroke researchers, stroke clinicians and paramedics. The game simulates a paramedic character, performing the standardised NIHSS assessment of a simulated patient. The order of the 11 different NIHSS observations is determined by the application and cannot be deviated from. The simulated patient was programmed based on 12 unique simulated cases presenting symptoms of acute stroke based on a wide variety of NIHSS scores resulting in an algorithm with over 1000 variations. The game is divided into three difficulty levels: easy, medium and hard to make the game evolve and increasingly demanding. GameStroke features a range of game elements introduced to create engagement. The players can choose one of four avatars or upload a profile picture for a more personalised experience. The score per completed case is based 70% on the right answer and 30% on time incentives. They receive rapid feedback with an overview of the examination, right and wrong answers, time use and total score at the end of each game. The players can track the leaderboard, providing a social and competitive element; this can however be opted out of by playing offline. They can also earn badges and advance to more challenging levels as they master the game. GameStroke uses pictograms (Fig. 1) developed for the ParaNASPP NIHSS application and validated against the standardised paper version [9]. The game also consists of educational elements to help students improve their skills. Assessment of the 11 items of NIHSS are explained in text and this information is accessible at all levels.

The development of the GameStroke application was conducted on an iOS platform and started in May 2020, and the beta version of the game was ready for testing in April 2021. A designated test group of stroke physicians, paramedics and third-year paramedic students explored the beta version of the game and provided feedback, until August 2021, when the final version was ready. The game is currently available by invitation only and was limited to the students enrolled in the study.

### Inclusion criteria

Paramedic bachelor students in their second year at Oslo Metropolitan University, Norway (OsloMet), were invited to participate. The study took place from allocation (September 3, 2021) until the clinical proficiency test (November 8, 2021).

All students who agreed to participate signed an electronic written consent form and provided necessary background information.

**Fig. 2:**
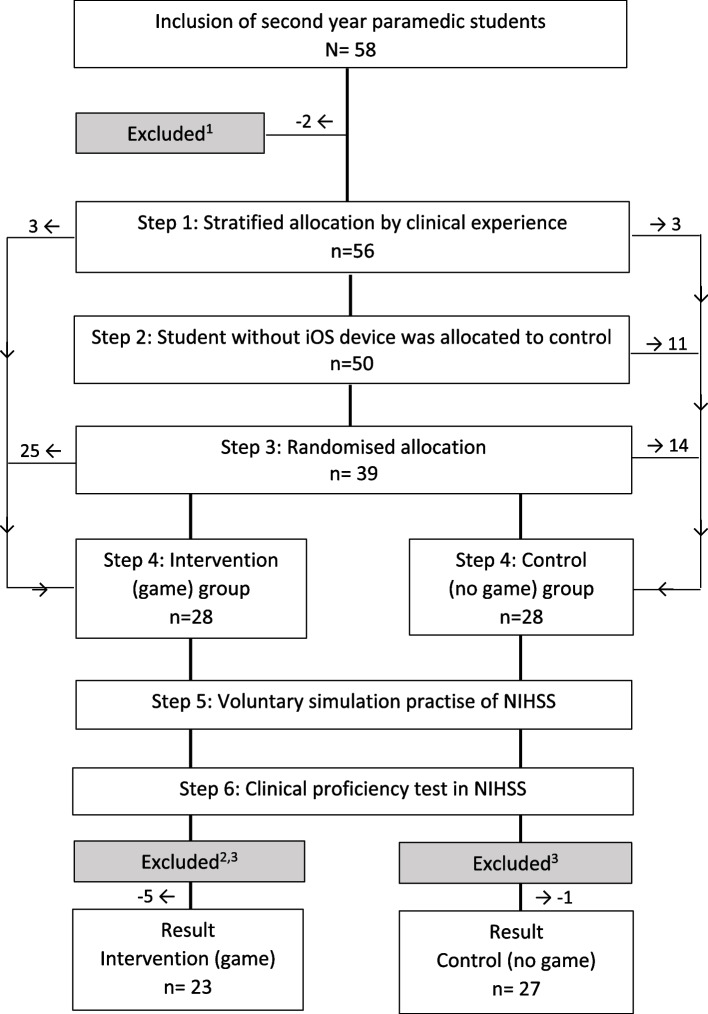
Study population. Superscript digit one (^1^) indicates the following: two students did not attend the mandatory lecture. Superscript digit two (^2^) indicates the following: four students did not download the intervention/application. Superscript digit three (^3^) indicates the following: two students did not take the clinical proficiency test

All enrolled students received a validated NIHSS training program for paramedics [7] consisting of a 45-minute lecture and a practical demonstration of the NIHSS examination by an NIHSS-certified physician, followed by a 45-min simulation session in smaller groups where they practised the examination on each other in pairs (Fig. 2).

### Step 1: Stratified allocation

Six students had previous relevant clinical experience which could potentially affect the study results. To secure equal allocation of these students in the two study groups, the students were paired based on the extent and similarity of their clinical experience.

### Step 2: Pragmatic allocation

Secondly, as the game currently only exists for iOS, students who did not own an iOS device were placed in the control group.

### Step 3: Randomised allocation

Finally, the remaining students were randomly assigned to either the game or control group using the web-based ‘Research Randomizer’ [18]. This was done by an outsider of the project employed at OsloMet.

### Step 4: Informing the groups

The control group received a link to the online self-report form and the game group received instructions on how to download the GameStroke application, using a two-factor authorization to validate the players’ identification.

### Step 5: Simulation training

NIHSS simulations training was voluntary for both groups. The control group was asked to fill out a short online self-report form if they did a simulation session to practice their NIHSS assessment skills. These simulations were arranged by the students without an instructor present. In addition to the number of simulations performed and time spent on each case, students in the control group reported the total amount of time spent simulating. This included time spent playing the role of ‘patient’ and observing fellow students’ examinations when waiting for their turn, since learning also takes place through observation [19].

For the game group, simulation activities including the number of completed games, time and date and time spent per simulation were automatically registered in the GameStroke application. The data collected was linked to the student’s mobile phone number.

### Step 6: Clinical proficiency test

**Fig. 3:**
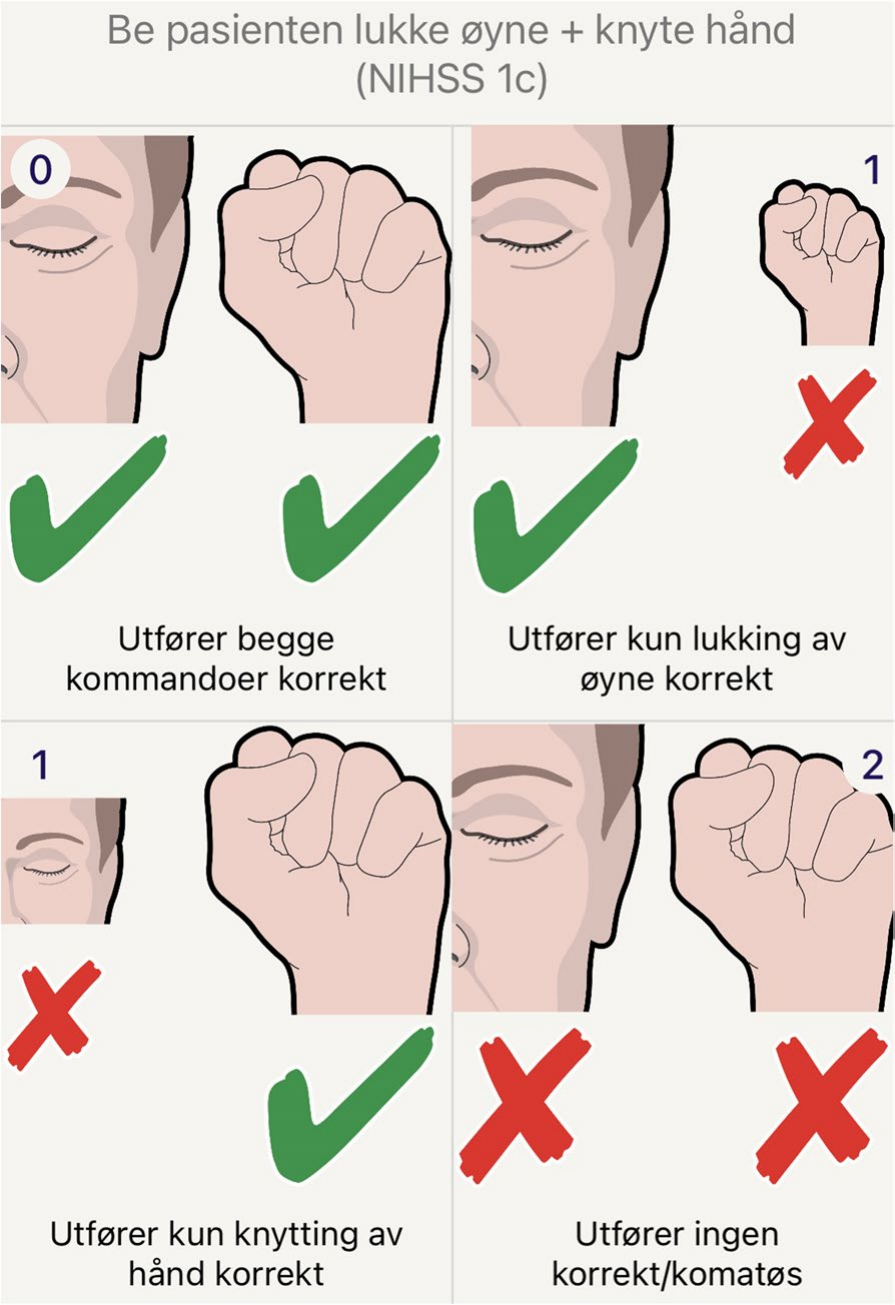
Pictogram of the NIHSS command 1c. In the clinical proficiency test, all students used a special form with pictograms, instead of the standard NIHSS text form

After the 2-month period of voluntary simulation training, all study participants completed a clinical proficiency test to assess their skills in scoring NIHSS. They were tested simultaneously by observing and scoring video recordings of six NIHSS assessments, where a paramedic examined a fictitious patient acting different scripted stroke cases with a pre-defined NIHSS score. The video recordings have been used in previous validation studies in the ParaNASPP trials and a slight variance (± 3) was deemed acceptable based on these results [7, 8]. In the clinical proficiency test, all students used a special form with pictograms, instead of the standard NIHSS text form. The pictograms (Fig. 3) were taken from the ParaNASPP application, which has shown very good agreement with the original NIHSS paper form [8].

### Exclusion criteria

To have comparable groups, students who did not attend the mandatory lecture were excluded prior to group allocation. Students in the intervention group who did not download the application or those who did not take the clinical proficiency test were excluded before analysing the results.

### Data protection

The study was approved by the Norwegian Centre for Research Data (reference no. 543238). The consent form and self-reported training log for the control group were created online using an encrypted version of the Tjenester for sensitive data (TSD), a research platform for sensitive data. Data for the game group was automatically collected by the application. The students in the game group also accepted GDPR consent when logging onto the GameStroke application for the first time.

### Statistics

Mann-Whitney *U* test was used to compare the skewed continuous variables in the game and control group, including differences in the number of simulations, time spent on simulation in total and time spent per simulation. Pearson’s chi-square test was used for categorical variables.

A Bland-Altman plot with 95% limits of agreement (LoA) [20] was used to assess the interrater agreement between the NIHSS scores in the game and control group, as well as between each of the two groups and the pre-defined score of each of the six films. Descriptive statistics and Mann-Whitney *U* tests were performed using SPSS V27 (IBM Corp, 2020). The Bland-Altman analysis was performed using R V4.0.3 (R Core Team, 2020).

## Results

Out of 58 invited participants, all students volunteered to participate, of which 50 were included for final analyses: 23 in the game group and 27 in the control group (Fig. 2). Of the total 50 participants, the majority, 37 (74%) had an iPhone (iOS platform). Six students reported previous clinical experience with stroke assessment. The students had an average (range) age of 23.3 (19 to 42) years and 28 (56%) were female (Table 1).

**Table 1:** Characteristics and description of the two groups and their NIHSS simulation

	Intervention (game) group (***n*** = 23)	Control (no game) group (***n*** = 27)
Female, *n* (%)	12 (52.2)	16 (59.3)
Age, mean	23.9 (SD: 4.83)	22.8 (SD: 2.51)
Median	22	22
Min–max	20–42	19–29
iOS users, *n* (%)	23 (100)	14 (51.8)
Student reported clinical experience of acute stroke examination, *n*	3	3
Average number of simulations, *n*^a^	14.4	2.47
Min–max	1–60	1–4
Percentiles^a^:		
25%	6	1
50%	10	3
75%	17	3
Average time spent per simulation^a^	2:57 min	3:50 min
	42:36 min (SD: 36)	9:28 min (SD: 8)
Average time spent on simulations^a^		28:36 min^b^
Percentiles^a^:		
25%	20:57 min	5 min
50%	34 min	30 min
75%	52:24 min	30 min

^a^Only the students who practised NIHSS are included (game group: 23 students, control group: 15 students)

^b^When including the time the students spent on their examination, observing fellow students and playing the role of the stroke patient

In the intervention group, 23 of the 28 students (82%) used the game application, whereas only 15 of the 27 (55.5%) in the control group self-reported that they had simulated (*p* < 0.003). There was a significant difference in the total time spent on simulations. The intervention group spent an average (SD) of 42:36 min (36) on gaming, whereas those who simulated in the control group spent 9:28min (8) simulating. In total, the game group spent 16.3 h on game simulation, compared to the control group who only spent 2.4 h on active in-person simulation (*p* < 0.000). When including time spent passively observing fellow students’ examinations or playing the role of the stroke patient, the control group spent a total of 7.1 h on in-person simulation, which is still significantly less than the game group (*p* < 0.000).

The intervention group completed a total of 333 games (average 14.4 per student, SD: 13), whereas those who simulated in the control group completed a total of 36 simulations (average 2.47 per student, SD: 1) (*p* < 0.000). The average time spent per simulation differed significantly, by almost a minute between the game group (2:57 min) and the control group (3:50 min) (*p* < 0.004).

**Fig. 4:**
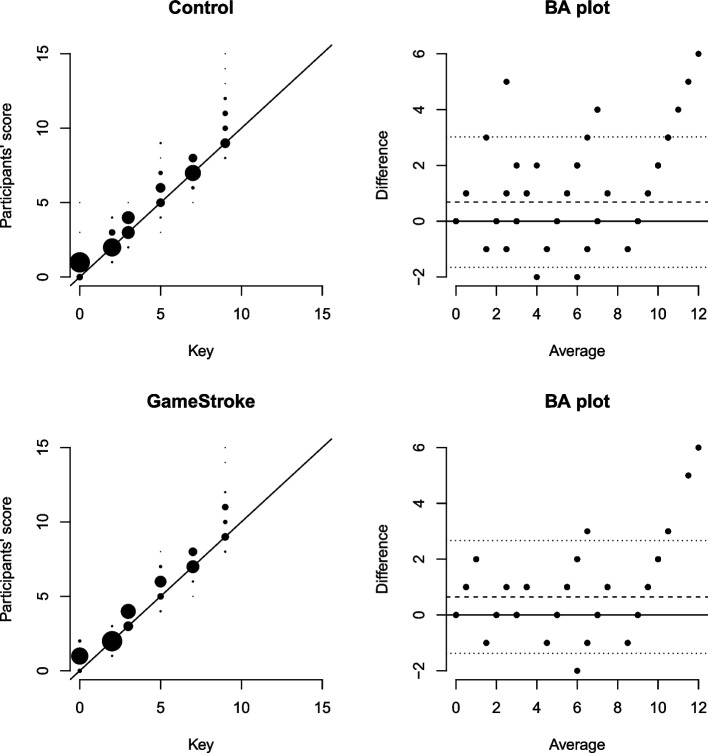
Bland–Altman plot of agreement of the clinical proficiency test according to group. The left graph: the line represents the scripted NIHSS score for each of the six videos (key). The students’ scores are shown as dots. The vertical distribution shows the range. The size of the dots represents the density of answers, i.e. how many students gave the same score. The right graph: The Bland-Altman plot represents the deviation from the film’s scripted score, illustrated as a baseline. The *Y*-axis shows how the students’ scores differ from the scripted score. The *X*-axis shows the average NIHSS scores given by the group

Bland-Altman analysis showed little difference between the two groups (Fig. 4). The largest deviation from the scripted NIHSS scores for the six videos, ranged from − 2 to 6, for both groups. Also, both tended to slightly overestimate the NIHSS from the scripted scores, with a mean difference of 0.64 (LoA: − 1.38 to 2.67) for the game group and a mean of 0.69 (LoA: − 1.65 to 3.02) for the control group, placing the LoA just over the acceptable ± 3 for the control group.

## Discussion

This study shows that gamification is a feasible training method for NIHSS assessment and leads to significantly more time spent on simulation training, compared to standard in-person simulation training. Both groups had a high level of agreement.

Traditional NIHSS simulation requires participants to attend in person [14], making it more time- and resource-demanding and more time is spent passively observing. We found that only around 1/3 of the time was an active simulation, compared to the 100% active participation time in the game group. The instant accessibility and availability of the game are probably the most important factors to achieve almost a tenfold increase in completed simulations in this group compared to the control. The GameStroke application was available anywhere, and at any time, on the student’s mobile phone. It can be assessed on the bus, in between lectures, on-call waiting for a dispatch—and is not limited to the availability of a mannequin simulating stroke symptoms. Furthermore, GameStroke may accommodate a surge of several hundred students in an instant, without the need for changes or preparation.

Although we found a major difference between the groups in the average number of simulations per student (14 vs. 2.5), this was not associated with a difference in interrater agreement when tested in the proficiency test. It seems that paramedics trained in NIHSS have a high level of accuracy, but tend to overestimate the NIHSS score slightly, as seen in recent studies comparing prehospital NIHSS to in-hospital scores [2, 7]. The student’s high accuracy in both groups might be attributed to the validated NIHSS training program [7]. However, the game group completed their assessment a minute faster than the control indicating that gamification may operationalise decision-making which results in a more efficient assessment, compared to traditional simulation. Further studies are needed to test if gaming reduces examination time in a clinical setting, as reduced prehospital delay may have substantial effects on patient outcomes [1, 5, 21, 22].

These results are consistent with several previous studies which have found that gamification often leads to increased use and engagement [15] but infrequently leads to improved outcomes [13, 17, 23, 24]. The paramedic students in the intervention group seemed to be motivated by the gamification of the simulated NIHSS examination, with more (27%) students engaging in simulation training. Gamification intrinsically motivates students by learning or mastering a skill, while some are extrinsically motivated by competition, achievement or recognition [17]. These engaging game elements, used to obtain and retain the player’s interest [17, 23, 25], are also known as motivational affordances [23]. It must be noted, however, that the effect of such games among healthcare professionals and students varies, as so does the usability and quality of the games, ranging from quizzes to computer games [12, 23, 24]. The quality of the studies also varies [12, 13, 15, 24, 26, 27], e.g small sample size, no control group, short duration, no objective measure or follow-up of knowledge retention.

Gaming may be used to standardise both NIHSS training of health personnel and clinical assessment of stroke patients. With limited access to in-person simulation training, gamification opens new ways to acquire clinical skills and to provide further education and skill retention. It can also reduce operating costs [14, 26], although this has yet to be established in other studies as it is rarely examined explicitly [27]. The benefits of gamification were magnified during the pandemic, as digital learning may continue regardless of governmental infection control legislation [28, 29]. Furthermore, digital interventions are scalable [17]. Future perspectives involve the training of all healthcare personnel involved in acute stroke management.

### Strengths and limitations

The major strength of the study is the use of a control group with active learning and simulation [13]. We also report the passive simulation training time for the control group, as to avoid underestimation of actual training. By using video-recorded examinations in the clinical proficiency test, the patient’s response remained constant, thereby strengthening the reliability of the study. It also reflects the attributes of the raters, rather than the person being rated [30].

The major limitations include the partial non-randomised design. The applications measure time spent down to the second, but there is likely a considerable margin of error in the self-reported times. Due to the use of video recordings, we were unable to test their NIHSS assessment time in the proficiency test. Technical difficulties may arise with application-based gamification, and a system for reporting errors has to be in place. It must be noted that this is a traditional Bland-Altman analysis [20] and does not take mixed effect models into account.

## Conclusion

Paramedic students can learn to accurately assess NIHSS through game-based simulation training. Gamification of NIHSS seemed to give an incentive to simulate considerably more and to perform the assessment faster, compared to standard in-person simulation training. This feasibility study opens for further research on gaming as clinical skill training for healthcare personnel assessing stroke patients.

## Data Availability

The datasets generated and analysed during the current study are not publicly available because individual students can be recognised in the anonymous dataset (e.g. age), but they are available from the corresponding author upon reasonable request.

## Abbreviations

EMS: Emergency medical services
FAST: Face Arm Speech Test
GDPR: General Data Protection Regulation
iOS: iPhone operating system
LoA: Limits of agreement
NIHSS: National Institutes of Health Stroke Scale
OsloMet: Oslo Metropolitan University
ParaNASPP: Paramedic Norwegian Acute Stroke Prehospital Project
SPSS: Statistical Package for the Social Sciences
TSD: Tjenester for Sensitive Data (A Research Platform for Sensitive Data)
